# Nonsurgical endodontic management of mandibular first premolar with incomplete root‐end resection

**DOI:** 10.1002/ccr3.3551

**Published:** 2020-11-29

**Authors:** Khaled Al‐Manei, Kholod Khalil Al‐Manei

**Affiliations:** ^1^ Unit of Endodontics Division of Oral Diseases Department of Dental Medicine Karolinska Institute Huddinge Sweden; ^2^ Division of Endodontics Department of Restorative Dental Science College of Dentistry King Saud University Riyadh Saudi Arabia

**Keywords:** cone‐beam computed tomography, failed apicoectomy, mineral trioxide aggregate, nonsurgical retreatment, periapical periodontitis

## Abstract

The complexity of the nonsurgical endodontic retreatment should be weighed against the benefit of surgical approach. This case report describes uncommon diagnosis and successful nonsurgical endodontic management of mandibular first premolar with a previous history of failed apicoectomy and incomplete root‐end resection using mineral trioxide aggregate and cone‐beam computed tomography.

## Graphical abstract

Nonsurgical root canal retreatment can be regarded as a valuable treatment option for teeth with failed apicoectomy and incomplete root‐end resection. Mineral trioxide aggregate can be a material of choice for the reobturation of cases with defective root‐end resection.

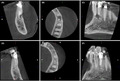

## 1 INTRODUCTION

The ultimate biological aim of endodontic therapy is to either prevent or heal apical periodontitis.^1^ It is well established that microbial infection of the root canal is the main cause of apical periodontitis.^2^ The presence of clinical signs and/or symptoms despite normal radiographical findings in the 2‐dimensional periapical radiograph could also indicate that apical periodontitis exists.^3^ However, the successful and predictable endodontic outcome is primarily governed by 3‐dimensional principles that consist of cleaning, shaping, and obturation of the root canal system.^4^

Root canal treatment can fail for various reasons, such as missing canals, ledges, canal transportation, instrument separation, and perforation.^5^ However, while endodontic therapy is performed under an aseptic working field and with adequate technical quality, the treatment can still fail due to other potential causes, including true cysts, foreign body reactions, accumulation of endogenous cholesterol crystals, extraradicular infection, or root fracture.^6^ Certainly, in well‐treated teeth, posttreatment apical periodontitis occurred in approximately 5%‐20% of teeth with preoperative apical periodontitis.^7^, ^8^

When primary root canal therapy fails, the treatment decision becomes more challenging to both the patient and the clinician. However, teeth with posttreatment apical periodontitis can be preserved and managed by either nonsurgical retreatment or endodontic surgery if the tooth is restorable and periodontally sound.^9^ Nonsurgical retreatment is commonly considered the first treatment choice to eradicate or reduce the microbial load, remove the infected root filling material, and disinfect and reobturate the root canal system.^10^ This retreatment procedure will create a favorable environment for periapical healing and a successful clinical outcome.^11^ Periapical surgery is an alternative treatment approach after unsuccessful orthograde retreatment or when orthograde retreatment becomes impractical or unfavorable.^12^ Nonetheless, several studies have reported that periapical surgery and nonsurgical retreatment have stable outcomes with 92% and 80% overall pooled success rates, respectively.^13^

At a lower rate, some previously treated cases with endodontic surgery exhibit unsuccessful outcomes, and posttreatment apical periodontitis is newly emerged or persisting.^14^ To address this dilemma, different treatment strategies should be suggested to the patient after a thorough and meticulous evaluation of the possible reasons for the unsuccessful previous treatment. Various treatment options have been proposed for the management of teeth with posttreatment apical periodontitis following endodontic surgery, including (a) extraction of the tooth with or without placement of a dental implant, (b) repeat surgery with or without previous or simultaneous retreatment of the coronal part of the root canal, and (c) nonsurgical retreatment with or without removal of the retrograde filling.^15^

The outcome of nonsurgical retreatment after unsuccessful apicoectomy has not attained much consideration in the endodontic literature. Several case reports have illustrated favorable outcomes following nonsurgical retreatment of failed apicoectomy.^14^, ^27^ In addition, a cohort study showed an 87% successful treatment outcome in 23 cases treated by orthograde retreatment and reobturated with mineral trioxide aggregate (MTA) after failed apicoectomy.^28^ However, to date, no study or case report has described the outcome of nonsurgical retreatment after failed apicoectomy in teeth with incomplete root‐end resection. Therefore, the present case report aims to describe the uncommon diagnosis and successful nonsurgical retreatment of mandibular first premolar with a previous history of failed apicoectomy and incomplete root‐end resection using cone‐beam computed tomography (CBCT).

## 2 CASE REPORT

A 64‐year‐old male patient with a noncontributory medical history was referred to the Endodontic Specialist Clinics for endodontic management of the lower left first premolar (tooth #34) with posttreatment apical periodontitis. The patient does not have a history of allergic reaction or current use of any medications. The patient had primary root canal treatment on tooth #34 approximately 7 years ago. Two years after the initial root canal therapy, the patient started to develop swelling, pain, and pus discharge. The referring dentist decided to perform apical surgery and to prescribe an antibiotic to the patient. However, the patient could not recall either the name of the antibiotic or the type of analgesic that he used. Following apical surgery, the patient was relieved from swelling with mild discomfort during biting.

**FIGURE 1:**
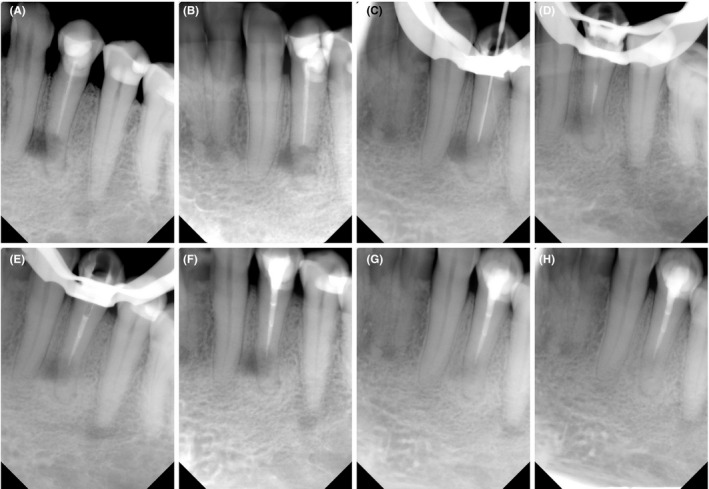
A, Preoperative periapical radiograph (straight angulation), B, preoperative radiograph (mesial angulation), C, working length radiograph, D, MTA apical plug radiograph, E, postobturation evaluation radiograph, F, postoperative periapical radiograph, G, 1‐y follow‐up radiograph, and H, 2‐y follow‐up radiograph

**FIGURE 2:**
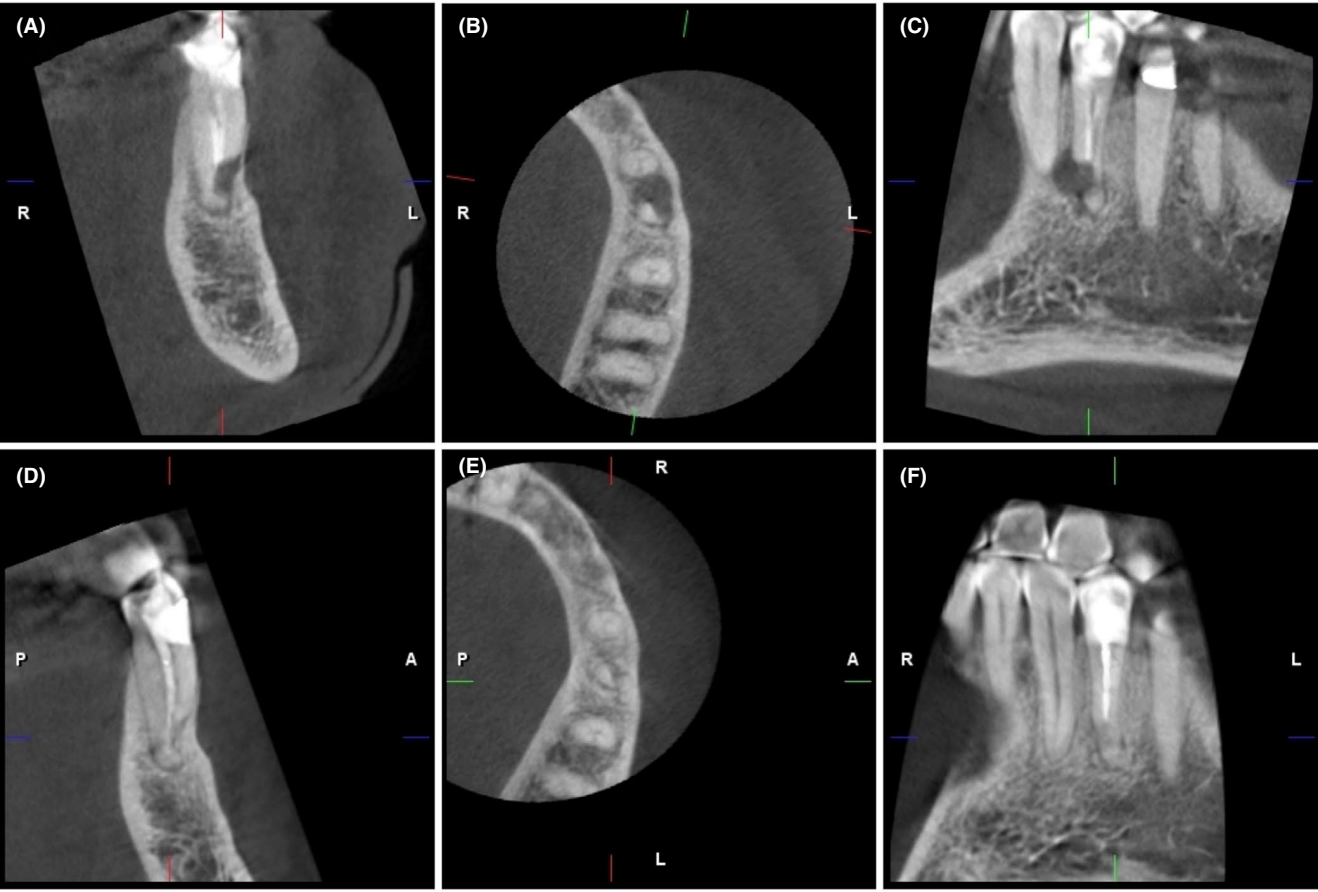
A, B, C, Preoperative coronal, axial, and sagittal CBCT images, and D, E, F, 1‐y follow‐up coronal, axial, and sagittal CBCT images

**FIGURE 3:**
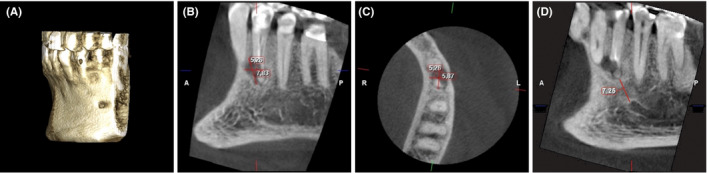
A, CBCT three‐dimensional image, B, C, extension of mesiobuccal radiolucency in the sagittal and axial images, and D, the distance from root apex of tooth #34 to mental foramen in sagittal CBCT image

Clinical examination revealed that tooth #34 had occlusal composite and cervical glass ionomer restorations with intact margins. There was no intra‐ or extraoral swelling, sinus tract, mobility, or deep probing depth detected around the tooth. Tooth #34 was tender to percussion and palpation. No abnormal clinical signs or symptoms were observed in the adjacent or opposing teeth. The preoperative radiographical examination was based on periapical radiographs and CBCT scans. Periapical radiographs showed lateral and periapical radiolucent lesions on tooth #34, overlapping the apical third of the root structure and associated with inadequate root canal filling (Figure 1A,B). A CBCT scan was obtained with a ProMax 3D MID (Planmeca Oy) operating at 90 kV and 10 mA with a small field of view (FOV) of 40 × 50 mm and a voxel size of 150 μm. The coronal and sagittal CBCT scanning images displayed incomplete root‐end resection of tooth #34 in which the surgical cut was limited to the buccal and mesial sides of the root without removal of the apical, distal, and lingual walls (Figure 2A,C). In addition, the axial and sagittal CBCT images revealed mesiobuccally located radiolucency with 5.25 × 6.15 × 7.80 mm in the buccolingual, mesiodistal, and occlusoapical maximum directions, respectively (Figure 3). The buccal cortical bone was thinned by the periapical pathosis without perforation (Figure 2A,B). The distance between the root end of tooth #34 and the mental foramen in the CBCT image was 7.25 mm (Figure 3D).

According to the collected data from the clinical and radiographical examinations, tooth #34 was diagnosed as previously treated with symptomatic apical periodontitis. Both nonsurgical and surgical endodontic treatment options were presented and discussed with the patient. The absence of adequate root canal filling led to the decision to perform nonsurgical root canal retreatment on tooth #34, and the patient agreed to participate and signed the consent form. The patient was scheduled for two treatment visits.

In the first visit, tooth #34 was isolated by a #9 rubber dam clamp and disinfected with 30% hydrogen peroxide (Unperoxide; Home Science Tools) and 0.5% chlorhexidine (MICROSHIELD Tincture; Schuelke) after the administration of the local anesthetic agent (2% xylocaine with epinephrine 1:80 000; Dentsply Sirona). Access cavity preparation was performed using long‐neck round carbide burs (Dentsply Sirona) in sizes 12 and 14. The gutta‐percha was removed mechanically with Gates‐Glidden burs (Dentsply Sirona) and hand nickel‐titanium (NiTi) files (Dentsply Maillefer). The working length was determined using the apex locater (Root ZX; J. Morita) and was confirmed by the periapical radiograph (Figure 1C). The canal was shaped with a WaveOne GOLD file (Dentsply Sirona), size 45, taper 05, as the third instrument size after the removal of the gutta‐percha. Copious irrigation of the canal was performed with 2.5% sodium hypochlorite (NaOCl; Clinix; Dental Sky) using a 27‐gauge notched open‐ended needle (Monoject® 3‐mL endo syringes; Safco Dental Supply) and agitation with EndoActivator (Dentsply Sirona) for 30 seconds.. Then, the canal was flushed with saline and irrigated with 3% ethylenediaminetetraacetic acid (EDTA; Tubulicid Plus Endo 4 oz; Global Dental Products) for 60 seconds. As a final irrigation protocol, the canal was rinsed with saline and irrigated with 2.5% NaOCl. The canal was dried with sterile paper points and dressed by calcium hydroxide paste (Ca[OH]_2_; Delian) using lentulo spiral (MediPros^®^; HIT). The access of the tooth was temporized with a double sealing technique using zinc oxide eugenol paste (Endomethasone N; Septodont) and intermediate restorative material (IRM Caps; Dentsply Sirona).

After 4 weeks (second visit), the patient became asymptomatic, not tender to percussion and palpation. The patient did not report any discomfort during biting. The tooth was reassessed under the same aseptic field of the first visit. Ca[OH]_2_ was removed by a similar chemomechanical preparation technique used in the first visit. The canal was dried with sterile paper points and obturated with gray MTA (Angelus) as an apical plug. MTA was mixed according to the manufacturer's instructions. MTA was carried to the canal with an MTA MAP System (Dentsply Tulsa Dental Specialties) and compacted apically with a DX Condenser (Dentazon) and sterile paper points. The extent and thickness of the MTA plug (4 mm) were confirmed radiographically (Figure 1D). The remaining part of the root canal space was filled with warm thermoplasticized gutta‐percha (Calamus, Dentsply Sirona) and AH Plus root canal sealer (Dentsply DeTrey) (Figure 1E). The access cavity was filled with composite resin (Filtek™ Z250 XT; 3M ESPE), and the patient's occlusion was checked. All retreatment procedures were performed with the aid of a dental operating microscope (OPMI pico; Zeiss) at 6× power magnification. After the treatment, a periapical radiograph was taken (Figure 1F), and the patient was scheduled for follow‐up visits.

At a 1‐year follow‐up, the patient was symptom‐free without reporting any biting discomfort. Tooth #34 had intact occlusal and cervical restorations, normal probing depth, and no tenderness to the percussion or palpation. The periapical radiograph showed reductions in the sizes of the lateral and periapical radiolucencies (Figure 1G). A small FOV CBCT (ProMax 3D MID; Planmeca Oy) was also taken to truly analyze healing after the treatment. The CBCT scan revealed progressive osseous regeneration over the resected buccomesial root surfaces with complete healing of the apical area around the root apex (Figure 2D,E,F). The patient attended the 2‐year follow‐up visit and continued to be asymptomatic with normal feelings during biting. The tooth was fully functional, with normal clinical findings. The periapical radiograph showed no suspicious radiolucency or recurrent apical periodontitis (Figure 1H).

## 3 DISCUSSION

Several cross‐sectional studies have demonstrated a high prevalence of posttreatment apical periodontitis in endodontically treated teeth.^29^, ^30^ In the case of posttreatment apical periodontitis, tooth preservation decisions could include either nonsurgical retreatment or endodontic surgical intervention.^10^ In the past 10 years, the outcome of nonsurgical retreatment has been reported with a high success rate of more than 75%.^7^, ^11^ Two systematic reviews with meta‐analysis compared the outcomes of nonsurgical retreatment cases to those with traditional or microsurgical endodontic treatment.^13^, ^31^ In the short‐term evaluation of the outcome (≤4 years), both traditional and microsurgical endodontic treatments had a more favorable success rate over nonsurgical retreatment.^13^, ^31^ Nonsurgical endodontic retreatment showed a higher healing rate than surgical endodontic treatment in the long‐term follow‐up (>4 years).^13^, ^31^ The outcomes of these treatment choices are typically affected by different clinical factors, such as the presence and size of the apical lesion, the root‐end filling material, iatrogenic errors, coronal restoration, and the status of previous root canal treatment.^7^, ^13^ Therefore, the patient should be aware of the potential prognostic factors that might reduce the success of each treatment option before proceeding with the treatment decision.

The present case report illustrates the successful nonsurgical retreatment of the mandibular first premolar with failed apicoectomy and incomplete root‐end resection. The recall periapical radiographs and CBCT scan appeared to suggest healing of the lateral and apical radiolucencies and bone regeneration in the area of incomplete root‐end resection. In this case, nonsurgical retreatment was selected as the first choice due to the lack of adequate root canal filling. In addition, the majority of posttreatment apical periodontitis is caused by sustained and colonized intraradicular bacteria rather than by extraradicular infection.^2^, ^6^ Although root‐end filling provides good sealing for the root canal space, leakage of the microbial infection after surgical treatment can cause redevelopment of apical periodontitis. Consequently, nonsurgical retreatment should be considered before any surgical procedure.^23^ Some of the patients are reluctant to be subjected to another surgical procedure. Undoubtedly, the second attempt of apical surgery may cause discomfort to the patient and may result in further scarring, bone loss, and compromising the crown‐to‐root ratio.^19^, ^21^ In the present case, a 2‐year follow‐up period was adopted, as most of the apical periodontist cases healed within this period.^32^ However, cases with large apical lesions may necessitate further follow‐up time to achieve complete healing.^7^

Several reports have been published on the nonsurgical retreatment of failed apicoectomy cases with successful treatment outcomes.^14^, ^27^ All the reported cases had a history of complete root‐end resection with or without proper placement of the retrograde filling material. Indeed, the endodontic treatment of these cases was built on the concept of treating the teeth with open apices or iatrogenic perforation. In some of the cases, the gutta‐percha was used for canal reobturation, while in the others, the MTA was selected to fill the canal space. In our case, the canal was reobturated by a 4‐mm MTA apical plug and backfilled with gutta‐percha. The MTA plug in the apical portion of the root stimulates periapical reconstruction by regeneration of the periodontal ligaments and formation of cementum‐like hard tissue.^33^ Although there is no consensus regarding the MTA placement techniques, the manipulation of the MTA apical plug in the current case was made with the aid of paper points to control compaction of the MTA in the apical direction, preventing any extrusion beyond the root‐resected area.^34^ Calişkan^16^ evaluated the nonsurgical retreatment outcomes of 11 treated teeth with failed apical surgery. Complete healing was observed in 5 cases (45.5%); however, MTA was not used as root‐end filling material. Furthermore, no information was mentioned regarding the use of the dental operating microscope during the retreatment procedure. In this case, the dental operating microscope was used throughout the treatment visits. Operating the endodontic cases under appropriate magnification can improve the success rate of the treatment.^28^ A study by Mente et al^28^ showed 87% successful orthograde retreatment outcomes of 23 cases with failed apicoectomy when the MTA was used as an apical plug. To the best of our knowledge, no data exist on the outcome of the orthograde retreatment of cases with failed apicoectomy and incomplete root‐end resection. The current case report is the first to describe effective nonsurgical management of a tooth with failed apicoectomy and incomplete root‐end resection using an MTA orthograde apical plug.

The success of nonsurgical endodontic retreatment relies on a proper disinfection protocol and adequate sealing of the root canal system.^11^ In this case, canal disinfection was achieved by abundant irrigation with 2.5% NaOCl and placement of a Ca[OH]_2_ root canal dressing. In a clinical study, the antimicrobial activity of 2.5% NaOCl was typically evident when the canal was enlarged to the third size instrument.^35^ The large apical preparation enhances the cleaning of the canal walls, allows more volume of irrigant fluid exchange, and leads to more disruption of the bacterial biofilm. Additionally, recent clinical studies have found no significant differences in the clinical or radiographical outcomes of endodontic treatment between 1% NaOCl and 2% chlorhexidine as the main irrigant or between the high (5.25%) and low (1%) concentrations of NaOCl.^11^, ^36^ However, the usage of NaOCl at high concentrations should be applied with caution to prevent the potential extrusion of NaOCl to the periapical tissues.^37^

In this case, the canal was dressed with Ca[OH]_2_ paste for 4 weeks. Ca[OH]_2_ is a commonly used intracanal medicament in endodontics due to its inherent alkalinity and antibacterial activity.^38^ It has long been acknowledged that the optimum antimicrobial effect of Ca[OH]_2_ in the clinical setting can be accomplished within 7 days of its application.^38^ However, it has been recently shown that neither 7 nor 14 days of Ca[OH]_2_ placement can improve canal disinfection.^39^ Following 30 days of Ca[OH]_2_ application, the levels of bacteria, pro‐inflammatory cytokines, and matrix metalloproteinases were significantly reduced in teeth with posttreatment apical periodontitis.^39^ While the high alkalinity of Ca[OH]_2_ has been suggested to lower the fracture resistance of teeth, no clinical studies have directly supported the association between Ca[OH]_2_ dressing and root fracture, regardless of the time of application.^40^ In fact, root fracture could be correlated with the stage of root development rather than the long‐term Ca[OH]_2_ dressing.^41^ Premedication of endodontically failed teeth with Ca[OH]_2_ has been associated with a more favorable clinical outcome.^42^ Moreover, dressing the canal with Ca[OH]_2_ improves the marginal adaptation of the MTA apical plug.^43^ EndoActivator was used in this case to aid the removal of the Ca[OH]_2_ paste from the canal. Some in vitro studies have shown superior performance of passive ultrasonic irrigation over EndoActivator or syringe irrigation in the removal of Ca[OH]_2_.^44^, ^45^ Certainly, none of the available endodontic irrigation techniques are capable of rendering the canal completely free from Ca[OH]_2_ remnants.^44^ Nonetheless, a contemporary systematic review highlighted that the use of ultrasonic activation did not affect the outcome of endodontic treatment when compared to hand syringe irrigation.^46^

A limited FOV CBCT is helpful in the diagnosis of periapical periodontitis and determination of the proximity to vital anatomical structures such as the mental nerve and mandibular canal.^47^ In the present case, CBCT was performed preoperatively to assess the extent of periapical radiolucency, explore the superimposed radiolucent area over the root structure, and evaluate the root canal anatomy. The CBCT images revealed incomplete root‐end resection, which could not be seen in the 2‐dimensional periapical radiograph. Defining the osteotomy site and precise root‐end resection can be achieved by a 3‐dimensional printed template for guided endodontic surgery.^48^ In addition, the distance between the root apex and the mental foramen was calculated to facilitate treatment planning and decision making. Previous studies have found that CBCT scans can alter the treatment plan in more than 50% of the total examined cases and change endodontic specialists' decisions, particularly in the retreatment of highly difficult cases.^49^, ^50^, ^51^ Furthermore, the CBCT scan has immense value in the assessment of healing after endodontic treatment.^47^ The postoperative CBCT images of the current case exhibited favorable osseous regeneration and bone remodeling around the root‐resected area. However, the CBCT scan implies the utilization of the X‐ray beam, such as any radiation dose, in which the dentist must abide by the ALARA (As Low As Reasonably Achievable) principle when using this technology. In the current case, the smallest FOV (40 × 50 mm) was also used as an equivalent to the radiation dose of 2 to 3 periapical radiographs.^52^ Nevertheless, acquiring the CBCT scan should be limited to complex cases with abnormal periapical findings.

In conclusion, careful assessment and treatment planning have a positive influence on treatment success. Nonsurgical root canal treatment is an effective treatment option in cases with incomplete root‐end resection and posttreatment apical periodontitis. MTA and other calcium silicate materials seem to be a material of choice for the reobturation of cases with incomplete root‐end resection. Further documented clinical cases with failed apicoectomy and incomplete root‐end resection with longer follow‐up time points are required to support this treatment modality.

## CONFLICT OF INTEREST

None declared.

## AUTHOR CONTRIBUTIONS

KA: participated in the clinical dental care of the patient. KA and KKA: contributed equally in the writing, literature review, and editing of the manuscript. The authors: read and approved the final manuscript.

## ETHICAL APPROVAL

Written informed consent was obtained from the patient for the publication of the text and images.

## Data Availability

Data sharing is not applicable to this case report as no datasets were generated or analyzed during the current report.
